# Supplementary material for: Bionic e-skin with precise multi-directional droplet sliding sensing for enhanced robotic perception
Source: Nat Commun. 2024 Jul 17;15:6022. doi: 10.1038/s41467-024-50270-8 (PMC11255283; doi:10.1038/s41467-024-50270-8)
Supplement: Supplementary file 1 — Supplementary Information [file 41467_2024_50270_MOESM1_ESM.pdf]

Supplementary Information

Bionic E-skin with Precise Multi-Directional Droplet Sliding Sensing for Enhanced Robotic Perception

Yunlong Xu[1,2,3], Zhongda Sun[2,3], Zhiqing Bai[4]*, Hua Shen[1], Run Wen[1], Fumei Wang[1], Guangbiao Xu[1]* and Chengkuo Lee[2,3]*

[1] Key Laboratory of Textile Science & Technology, Ministry of Education, College of Textiles, Donghua University, Shanghai 201620, China.

[2] Department of Electrical & Computer Engineering, National University of Singapore, 4 Engineering Drive 3, Singapore 117583, Singapore.

[3] Center for Intelligent Sensors and MEMS, National University of Singapore, 4 Engineering Drive 3, Singapore 117583, Singapore.

[4] Key Laboratory of Multifunctional Nanomaterials and Smart Systems, Suzhou Institute of Nano-Tech and Nano-Bionics, Chinese Academy of Sciences, Suzhou 215123, China.

Supplementary Notes

Supplementary Note 1 | The advantages of co-layer interlaced electrode networks.

As shown in Supplementary Fig. 3, both X-electrodes and Y-electrodes of co-layer electrode networks are on one layer without an additional spacer layer between them, so that the distance of charge induction between the triboelectric layer surface and each electrode is consistent. Other structures without co-layered electrode networks were designed by incorporating a spacer layer between the two series of electrodes. This structure will lead to an incompatibility in the distance between electrodes and the triboelectric layer, which can reduce sensitivity. Supplementary Fig. 12 shows the electrical signal output of the DES without the co-layer electrodes and overpass micro-connection structures. The difference in the signal values between the X- and Y-electrodes is significant due to the inconsistent charge induction distances between electrodes and the triboelectric layer.

In addition, the co-layer electrode networks using the overpass connection technology can also avoid area overlapping of two series of electrodes and greatly reduce the signal crosstalk. Supplementary Fig. 12 also shows that obvious crosstalk occurs in both dual-channel and multi-channel signals for the device without co-layer interlaced electrode networks.

Supplementary Note 2 | The Characteristics of the water droplet sliding along X-direction on DES.

The dual-channel electrical signals show that four electrical signals were output from each Y-electrode and three electrical signals were output from X_2 electrode (Supplementary Fig. 13 b). It means that the droplet passes through the X_2 electrode three times and the Y-electrodes four times. In particular, the multi-channel signals (Supplementary Fig. 13 c) show that three electrical signals are all output from the X_2 electrode in different time sequences, while the other four electrical signals are generated sequentially on the Y_1, Y_2, Y_3, and Y_4 electrodes, respectively. Based on the previous discussion, it can be determined that the droplet slides along the trajectory $Y_1 \rightarrow X_2 \rightarrow Y_2 \rightarrow X_2 \rightarrow Y_3 \rightarrow X_2 \rightarrow Y_4$.

Supplementary Figures

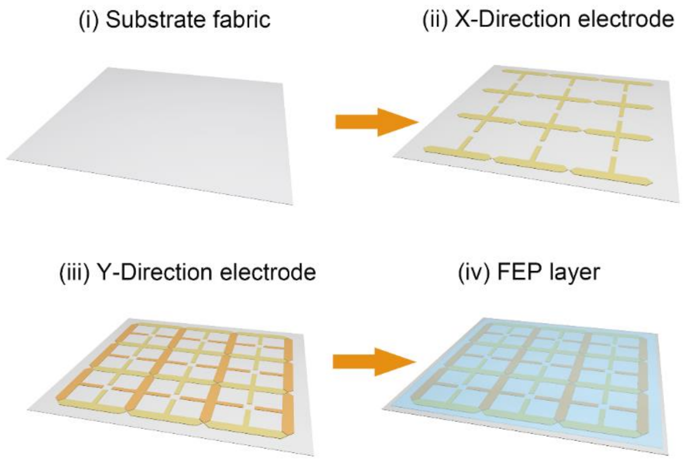

Supplementary Fig. 1 | Schematics of the preparation process of DES (Droplet e-skin).

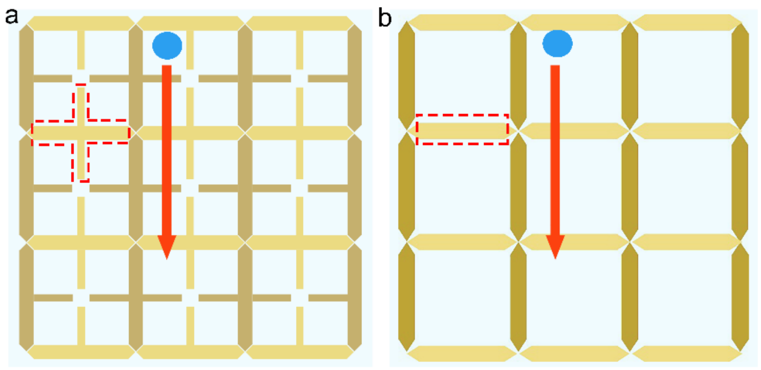

Supplementary Fig. 2 | The electrode networks (a) with branched electrodes and (b) without branched electrodes. The electrode coverage can be nearly doubled by branching structure in the co-layer interlaced electrode network without increasing the number of channels.

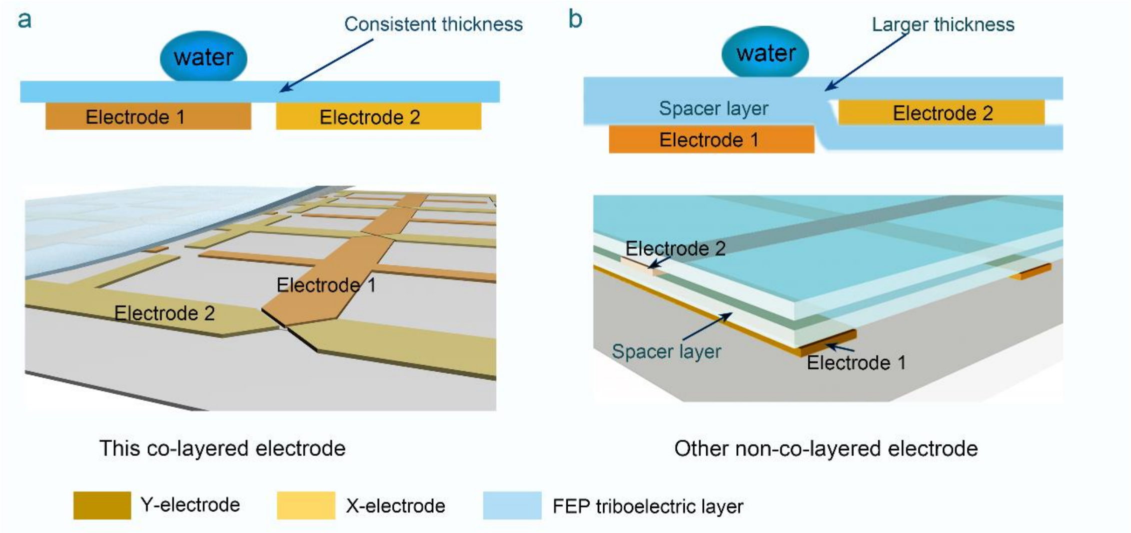

Supplementary Fig. 3 | Comparison of co-layer electrode (a) and non-co-layer electrode (b). The co-layer electrode with more consistent and thinner triboelectric layers.

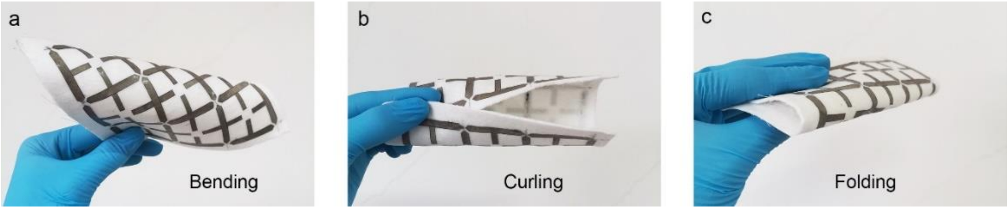

Supplementary Fig. 4 | The excellent flexibility of DES. DES can be easily bent (a), curled (b) and folded (c).

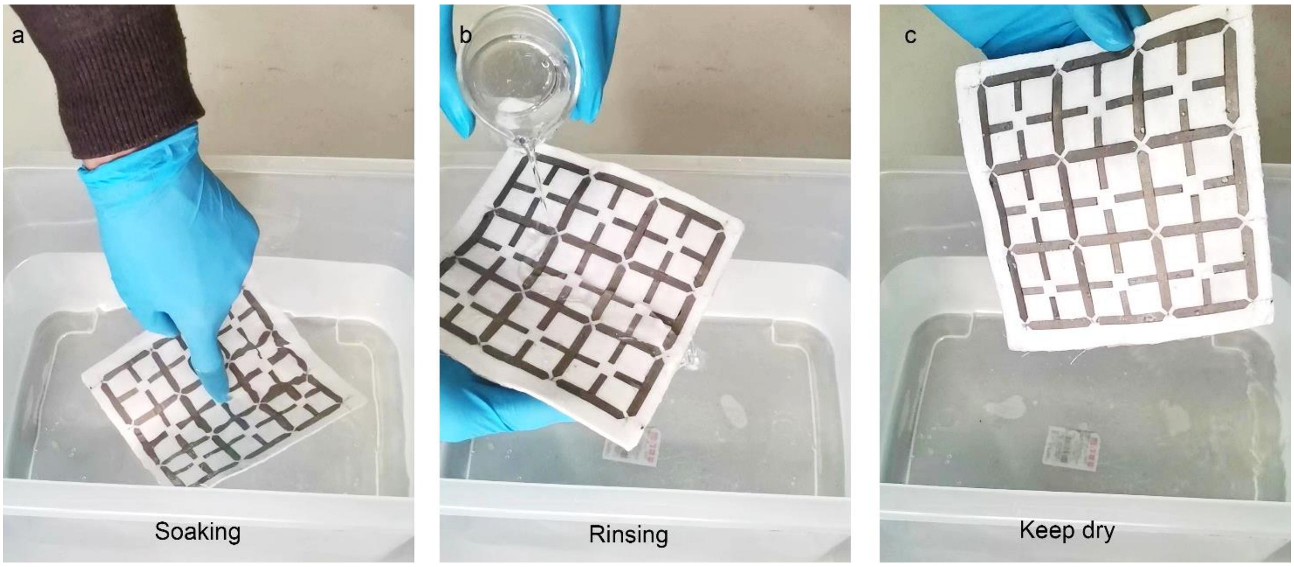

Supplementary Fig. 5 | Waterproof performance of DES. (a) DES is immersed in water. (b) DES is rinsed with water. (c) DES keeps dry after being soaked and rinsed.

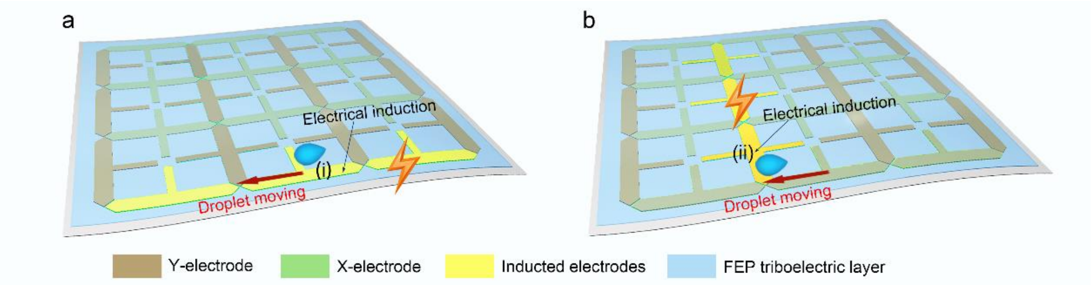

Supplementary Fig. 6 | State of charge induction of the DES as droplet slides from electrode (i) to electrode (ii).

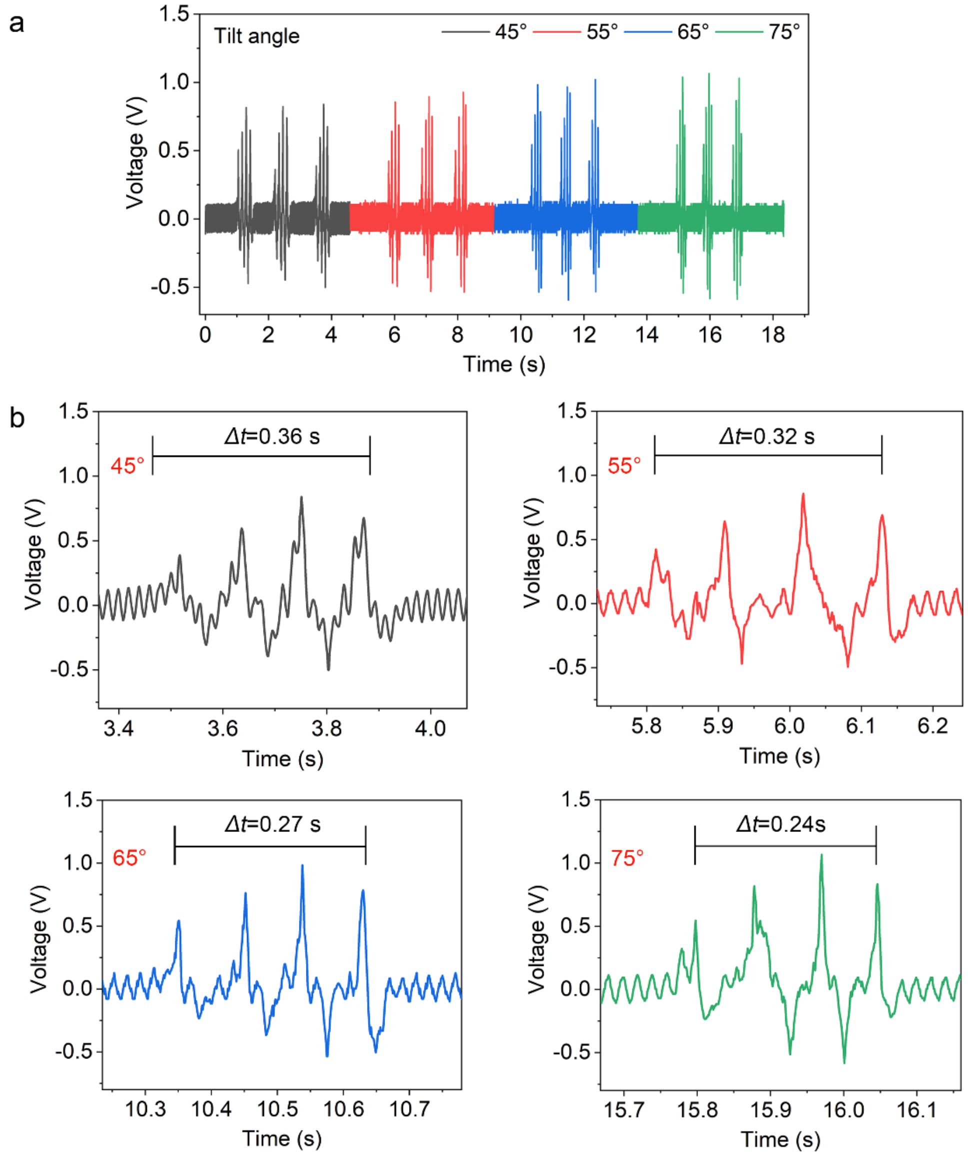

Supplementary Fig. 7 | Electrical output of DES at different surface angles (45°, 55°, 65°, and 75°).

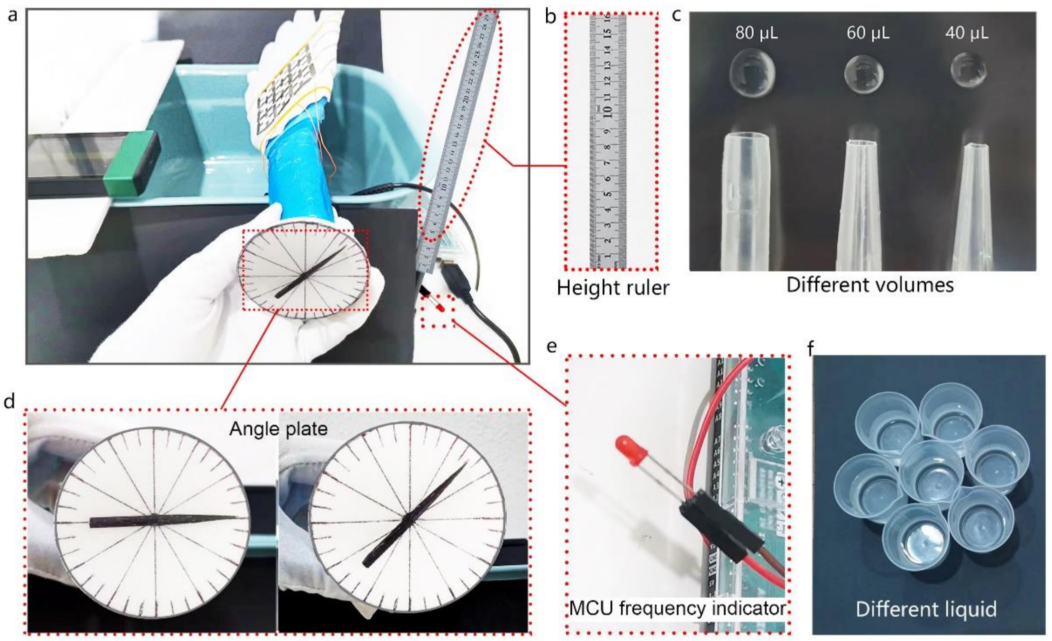

Supplementary Fig. 8 | (a) Measurement platform for droplet parameter detection. (b) Height scale ruler. (c) Droppers with different diameters. (d) Angle plate. (e) MCU (microcontroller units) frequency indicator. (f) Different kinds of liquid.

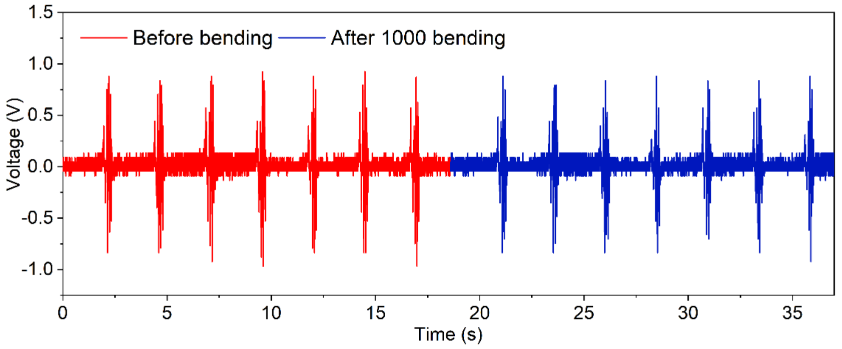

Supplementary Fig. 9 | The electrical output of DES before and after 1,000 repetitions of 90° bends.

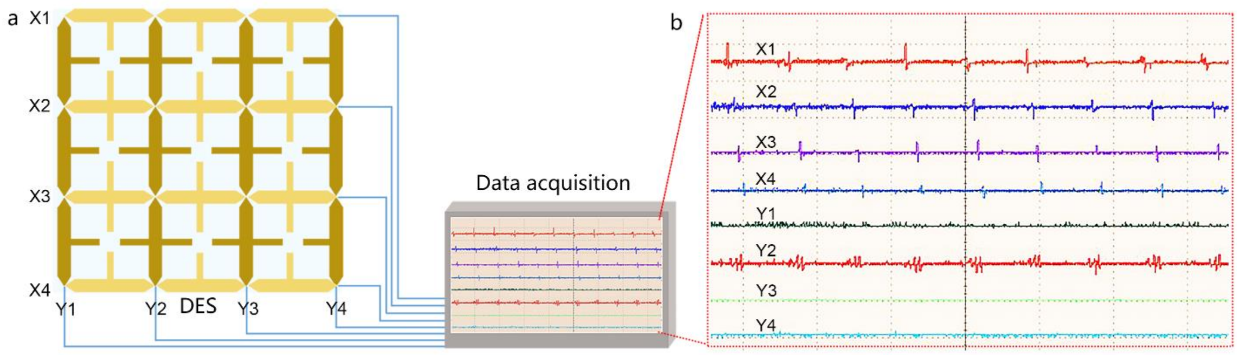

Supplementary Fig. 10 | Schematic of a multi-channel signal acquisition system for DES . (a) DES and (b) Multichannel electrical output signals.

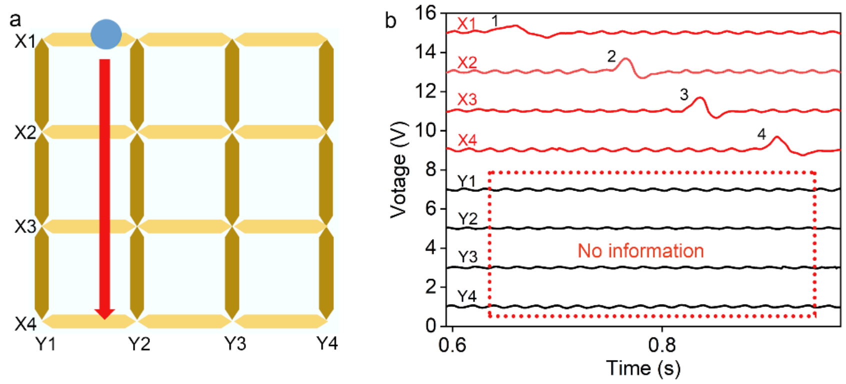

Supplementary Fig. 11 | Electrical signal of DES without branched electrodes**.** (a) Droplets sliding in the Y direction on DES without branched electrodes and (b) the multi-channel signals. The trajectory of the droplet cannot be analyzed from the electrical signal. It can only be determined that the droplet moves approximately in the Y-direction, without determining which Y-electrode it is near.

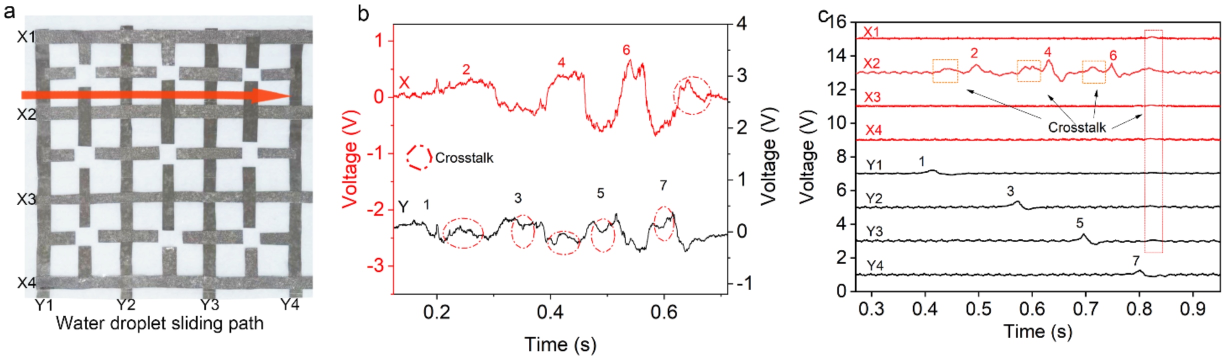

Supplementary Fig. 12 | The crosstalk validation of the DES without the co-layer interlaced electrode and overpass connection. (a) Schematic diagram of a droplet sliding in the X-direction. (b) The dual-channel signal. (c) The multichannel electrical signal all show obvious crosstalk.

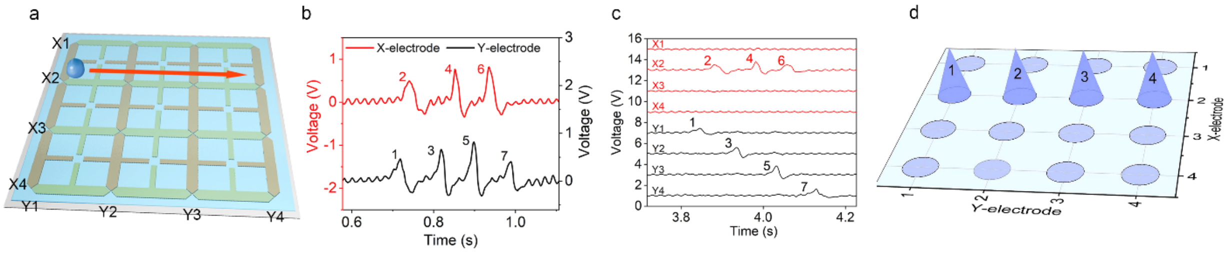

Supplementary Fig. 13 | The Characteristics of the water droplet sliding along X-direction on DES. (a) Schematic diagram of a droplet sliding in the X-direction. (b, c) The output voltages, and (d) the trajectory map.

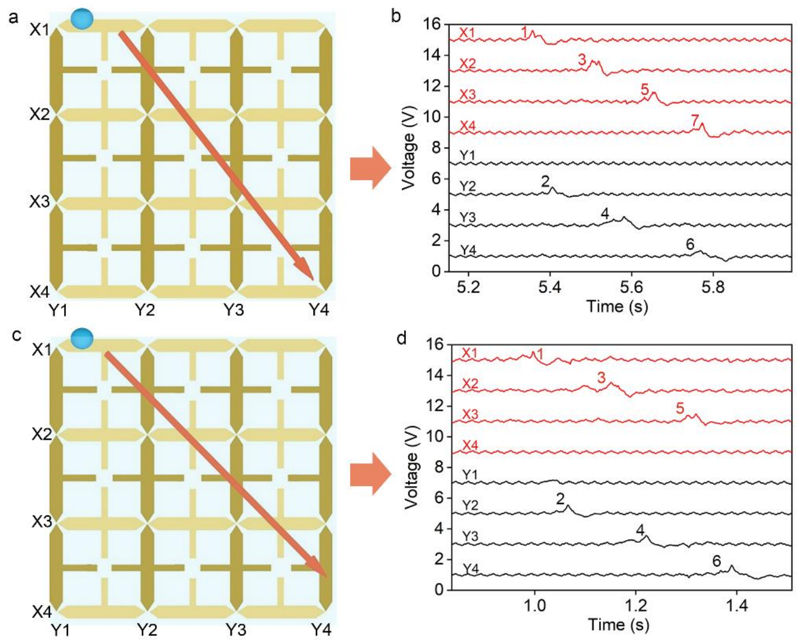

Supplementary Fig. 14 | The Characteristics of the water droplet sliding along oblique direction on DES. (a, c) The droplet sliding along different oblique direction and (b, d) the corresponding multi-channel electrical signals.

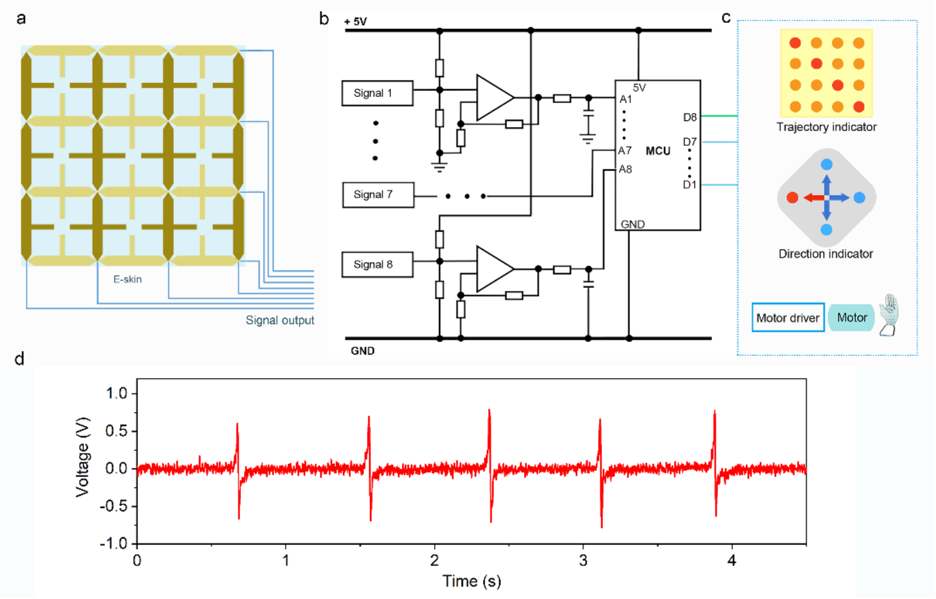

Supplementary Fig. 15 | The Architecture of the intelligent monitoring system. (a) DES (E-skin) with 8-channel output. (b) Signal processing circuits and microcontroller that used in the application. (c) Trajectory indicator, sliding direction indicator and motor driver for applications. (d) Voltage signal output by the signal processing circuit.

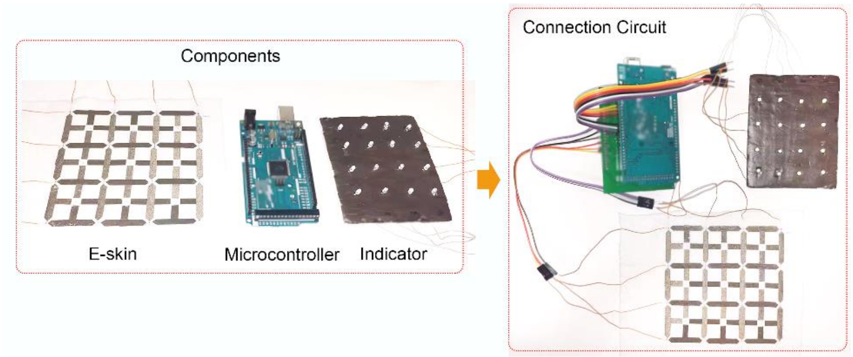

Supplementary Fig. 16 | The components and connections of the motion trajectory feedback system.

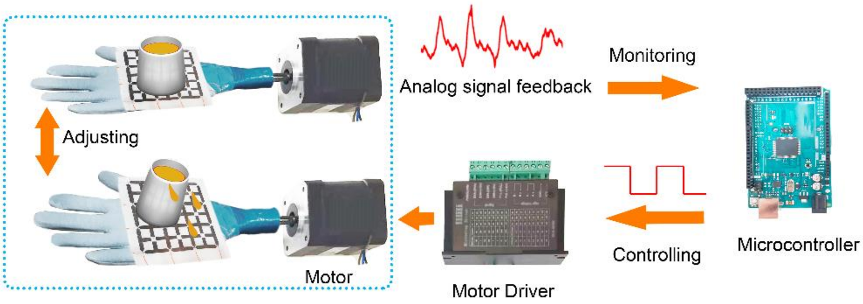

Supplementary Fig. 17 | Components and mechanisms of closed-loop control systems.
