# Supplementary material for: Bionic e-skin with precise multi-directional droplet sliding sensing for enhanced robotic perception
Source: Nat Commun. 2024 Jul 17;15:6022. doi: 10.1038/s41467-024-50270-8 (PMC11255283; doi:10.1038/s41467-024-50270-8)
Supplement: Supplementary file 3 — Description of Additional Supplementary Files [file 41467_2024_50270_MOESM3_ESM.docx]

**Description of Additional Supplementary Files**

**File Name: Supplementary Movie 1
Description:** Waterproof performance of DES.

**File Name: Supplementary Movie 2
Description:** Sensitivity and droplet parameter detection of DES.

**File Name: Supplementary Movie 3**

**Description:** Droplet motion trajectory tracking and visual feedback.

**File Name: Supplementary Movie 4**

**Description:** Droplet flow direction monitoring and warning.

**File Name: Supplementary Movie 5**

**Description:** Closed-loop control system for liquid leakages
